# Comparison of the effects on the pharyngeal airway space of maxillary protraction appliances according to the methods of anchorage

**DOI:** 10.1186/s40902-017-0101-9

**Published:** 2017-01-25

**Authors:** Won-Gyo Seo, Se-Jin Han

**Affiliations:** Department of Oral and Maxillofacial Surgery, College of Dentistry, Dankook University, 119 Dandae-ro, Dongnam-gu, Cheonan, South Korea

## Abstract

**Background:**

The purpose of the study is to compare the effects on the pharyngeal airway space of skeletal anchored face mask with those of tooth-borne facemask.

**Methods:**

We used two types of facemask for maxillary protraction, the tooth-borne facemask (TBFM) and the skeletal anchored facemask (SAFM), and evaluated the effects of each facemask on the pharyngeal airway. Twenty-eight patients (mean age 10.3 years) were treated with the TBFM and 24 patients (mean age 11.2 years) were treated with the SAFM. Lateral cephalometric radiographs were taken before treatment (T1) and after treatment (T2) to assess changes in the dimensions of the upper airway. Statistical analysis was performed with independent *t* tests, matched *t* tests, Mann-Whitney *U* tests, and Kruskal-Wallis tests.

**Results:**

There were marked increases in upper airway dimensions in both groups following treatment, but the SAFM group had a significantly greater increase in airway dimensions than the TBFM group. Also, the SAFM subgroups showed more improved airway measurements than the TBFM subgroups in both the superior and inferior pharyngeal airways.

**Conclusions:**

SAFM is more effective than TBFM in increasing upper airway dimensions.

## Background

Patients with skeletal class III malocclusions have a prominent and protrusive lower face and a relatively inconspicuous and retruding upper face, resulting in a concave profile. Class III malocclusions, which are skeletal facial deformities, are characterized by retrusion and deficiency of the maxilla and excessive growth and protrusion of the mandible. Almost two thirds of skeletal class III malocclusions are due to either retrusion of the maxilla or a combination of maxillary retrusion and mandibular protrusion [1–3].

The prevalence of class III malocclusions is only 1 to 3% in the Caucasian population. However, the incidence is as high as 14% in Asian populations [4–6].

Class III malocclusions are one of the most challenging orthodontic problems for practitioners to correct. The treatment modalities for skeletal class III malocclusions are growth modification for young patients and orthognathic surgery for adult patients. Growth modification treatment should start in earlier ages than treatment for other orthodontic problems, usually in the primary dentition or early mixed dentition stages [7, 8], so the entire treatment time can be extensive.

There are many maxillary protraction appliances for skeletal class III malocclusion which have been used since 1960 [9]. Numerous studies have investigated the dentoalveolar and skeletal effects of maxillary protraction appliances [10–12]. These appliances stimulate sutural growth of the maxillary complex by pulling the complex forward. This results in maxillary protrusion, clockwise rotation of the mandible, counterclockwise rotation of the palatal plane, proclination of the upper incisors, and retroclination of the lower incisors. However, the tooth-borne anchorage facemask (TBFM) has some disadvantages. The protraction forces of maxillary protraction appliances are applied to the first maxillary molar, so the effects are more dentoalveolar than skeletal in nature. This results in a relatively high rate of relapse to reverse overjet after the mandible is completely grown and dental side-effects, such as mesial migration and extrusion of the maxillary molars and labioversion of the maxillary incisors. The optimal age at which to begin treating patient with facemasks is somewhat controversial, but it is usually done at the stage of primary dentition or early mixed dentition [7, 8].

To address the disadvantages of the tooth-borne facemask, the skeletal anchored facemask (SAFM) was invented [10–12]. This appliance obtains stable anchorage in the facial bone and applies direct force to sutures in the maxillary complex.

Recently, many studies have revealed the efficacy of the SAFM compared to the TBFM, focusing on cephalometric measurements [13–15]. Also, there have been studies on the maxillary protraction effects of such facemasks on pharyngeal airway dimensions [16–18]. However, there are few articles comparing the effects of the SAFM and the TBFM on pharyngeal airway dimensions.

This study compares the effects of the SAFM and TBFM on the pharyngeal airway.

## Methods

Consecutive subjects were recruited from a private orthodontic clinic in Chunan, South Korea. Twenty-eight patients (8 boys and 20 girls) were treated with a TBFM and 24 patients (12 boys and 12 girls) were treated with an SAFM. All subjects had skeletal and dental class III malocclusions with an anterior crossbite or incisor edge-to-edge relationship and class III molar relationships, cervical vertebra maturation stage of 3 or 4. No patient had a congenital deformity with involvement of cervical vertebrae 3 and 4. No previous orthodontic treatment was performed on any of the patients. The initial mean age of the TBFM group was 10.3 ± 1.4 years (range, 8.4–12.6 years). The initial mean age of the SAFM group was 11.2 ± 1.1 years (range, 9.1–13.3 years). The mean duration of TBFM treatment was 14.3 months and that of SAFM treatment was 16.9 months.

Both the TBFM and SAFM groups were divided into subgroups A and B according to degree of maxillary protraction. Distance of condylion to A point (Co-A) and SNA angle were measured. Both measurements showed similar distributions in the TBFM and SAFM groups, so Co-A distance was chosen as the criteria for dividing patients into subgroups. Subgroup A included subjects who had a degree of maxillary protraction greater than the mean. Subgroup B had less maxillary protraction than the mean value. For example, SAFM subgroup A consisted of subjects whose maxillary protraction was greater than mean value of the SAFM group. The correlation between maxillary protraction and pharyngeal airway increase was studied by analyzing these subgroups. The protocol were approved by the Institutional Review Board (IRB) of Dankook university dental hospital (IRB No. H-1509/008/002), and this study followed the guidelines of Helsinki Declaration.

1. SAFM surgical protocol
  Anesthesia methods can vary from simple local anesthesia to intravenous sedation or general anesthesia. Usually, local anesthesia is sufficient, but younger patients and those with dental phobias sometimes require more advanced anesthesia. Once anesthesia was given, two incisions were made with a sharp scalpel or electrocautery in the anterior vestibule of the maxilla from the central incisor to the canines on both sides. A mucoperiosteal flap was elevated by blunt dissection to expose the anterior surface of the maxilla. Two curvilinear miniplates with 1.5 mm thick Synthes (Zuchwil, Switzerland) were placed in the lateral nasal walls of the maxilla on both sides of the aperture piriformis, superior to the apex of the canines on both sides. These two curvilinear miniplates were locking plates with eight holes. The plates needed modification preoperatively, which involved adjusting the plate length to seven holes or six holes and modifying the last hole into a hook to enable hanging of elastics from the facemask. Four locking screws (1.5 mm diameter, 6 mm length) were used to fix each miniplate. Usually, plate bending before placement was required. The reason for using a locking screw and plate system is that, as the patients are young, their bones are quite soft and flexible, so a conventional plate and screw system would not provide sufficient resistance to the elastic force of the facemask. After plate fixation, sutures were created with non-resorbable nylon. The hook-shaped end should be placed on keratinized gingiva to minimize irritation. Stitches were taken out 1 week after surgery. Application of the face mask was begun 2 to 3 weeks after the surgery. Patients were asked to wear the face mask all day long and the protraction force of the elastics was adjusted to 400–500 g on each side (Fig. 1).

**Fig. 1:**
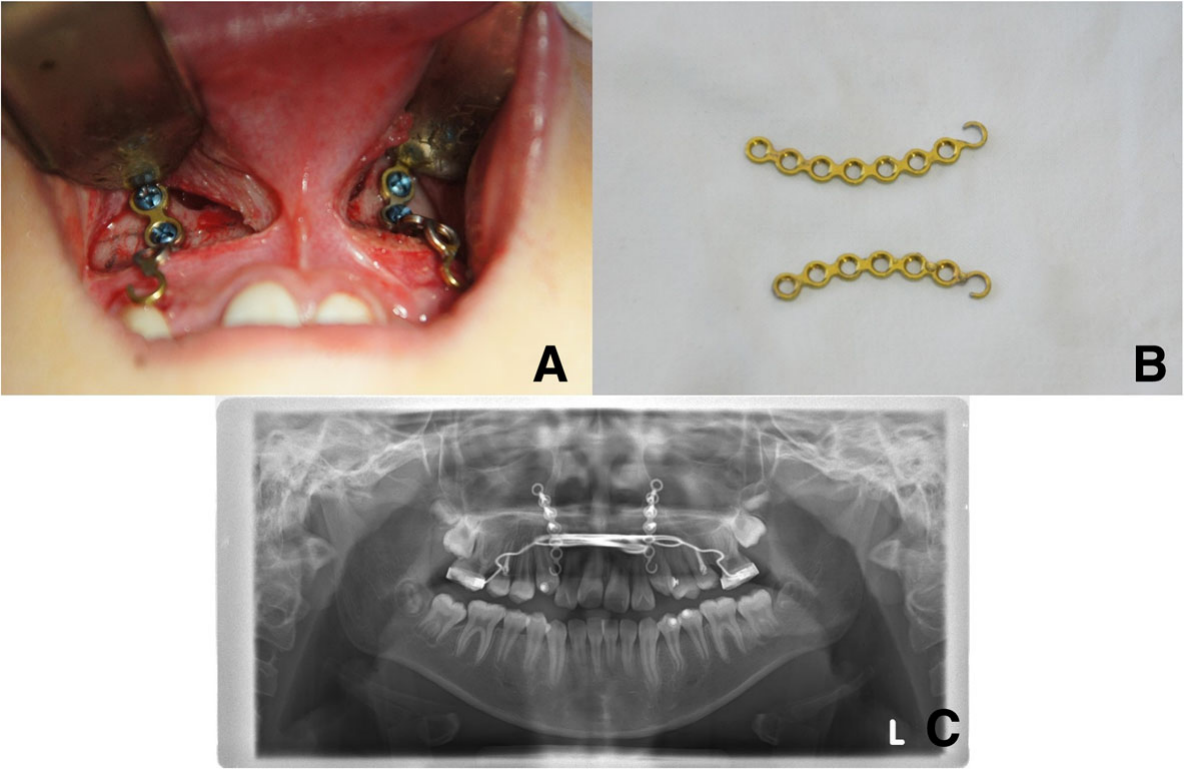
**a** Intraoral view of the application of two curvilinear plates on the lateral nasal walls of the maxilla. **b** Preoperatively modified miniplates (Synthes, Zuchwil, Switzerland). **c** Postoperative panoramic radiograph showing miniplate positioning
2. TBFM protocol
  Patients in the TBFM group received conventional face mask therapy and rapid palatal expansion therapy, in which the first maxillary premolars and first molar were banded with hooks on both sides. Patients were instructed to activate the palatal expander one or two times a day until slight overexpansion was obtained. Patients in the TBFM group were asked to wear the face masks for at least 12 h a day. Approximately 400 g of elastic force was applied on each side (Fig. 2).

**Fig. 2:**
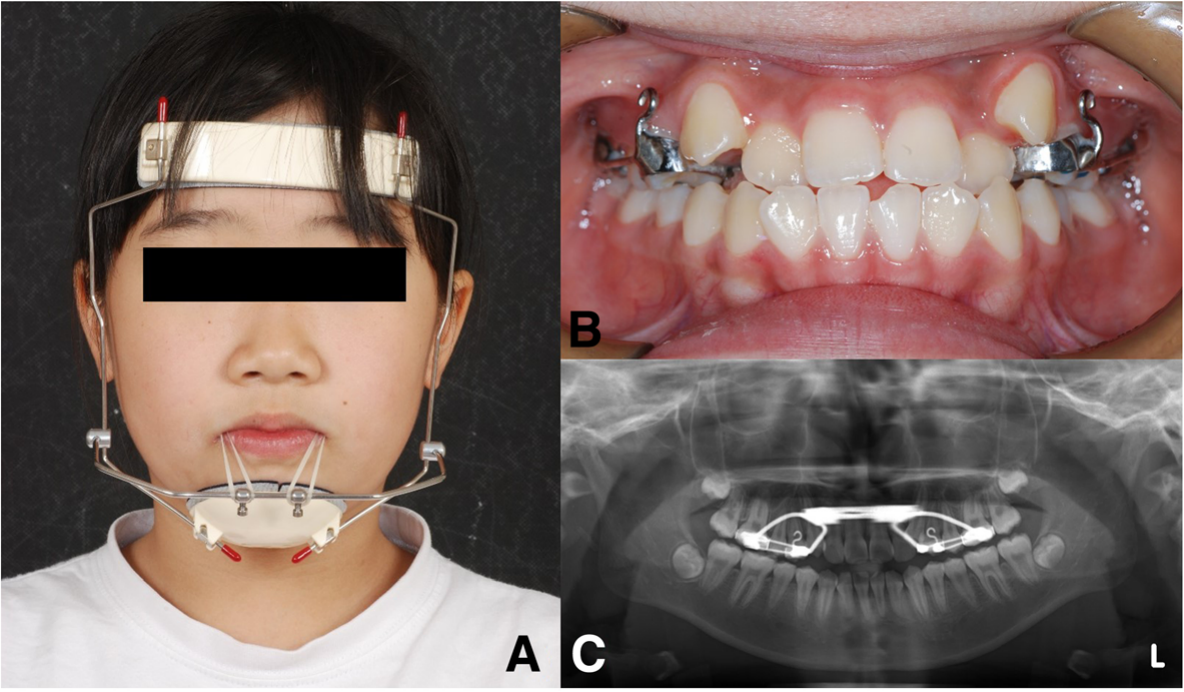
**a** Frontal view of the patient with a facemask. **b** Intraoral view of face mask and rapid palatal expansion. **c** Panoramic radiograph showing rapid palatal expansion

### Cephalometric analysis

**Fig. 3:**
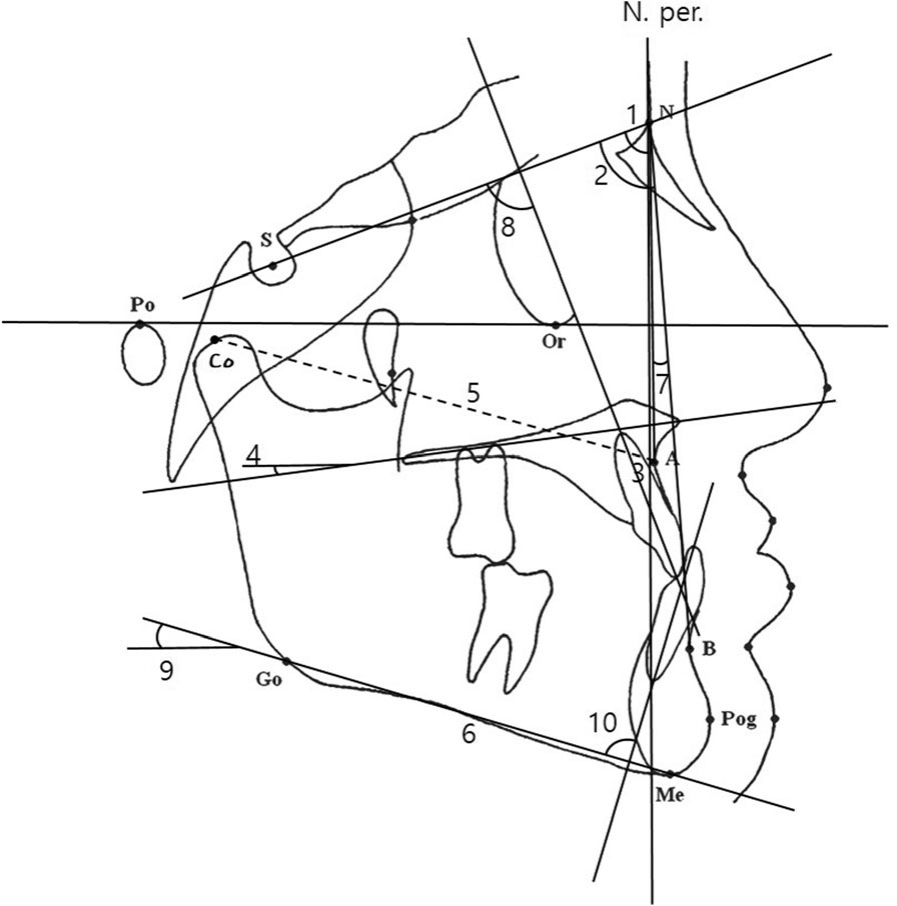
Cephalometric measurements used in this study. *1* SNA, *2* SNB, *3 N. Per.* to A, *4* palatal P, *5* Co-A (*dotted line*, maxillary advancement measurement), *6* Mn. Length, *7* ANB, *8* U1 to SN, *9* FMA, *10* IMPA

**Fig. 4:**
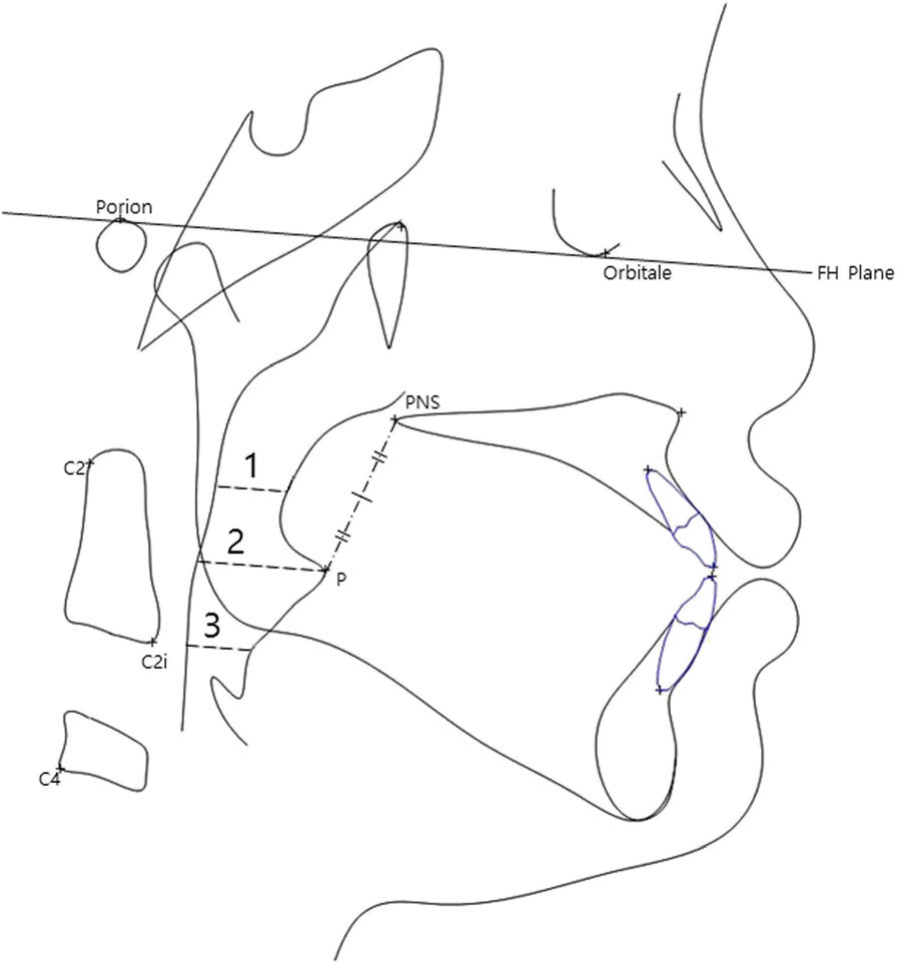
Anatomic points and linear measurements used in this study. *P* indicates the tip of the soft palate, *C2* the most posterosuperior point on the odontoid process of the axis, *C2i* the most anteroinferior point on the body of the second cervical vertebra, *C4* the most posteroinferior point on the body of the fourth cervical vertebra. *1* SPPS, *2* MPS, *3* IPS

**Fig. 5:**
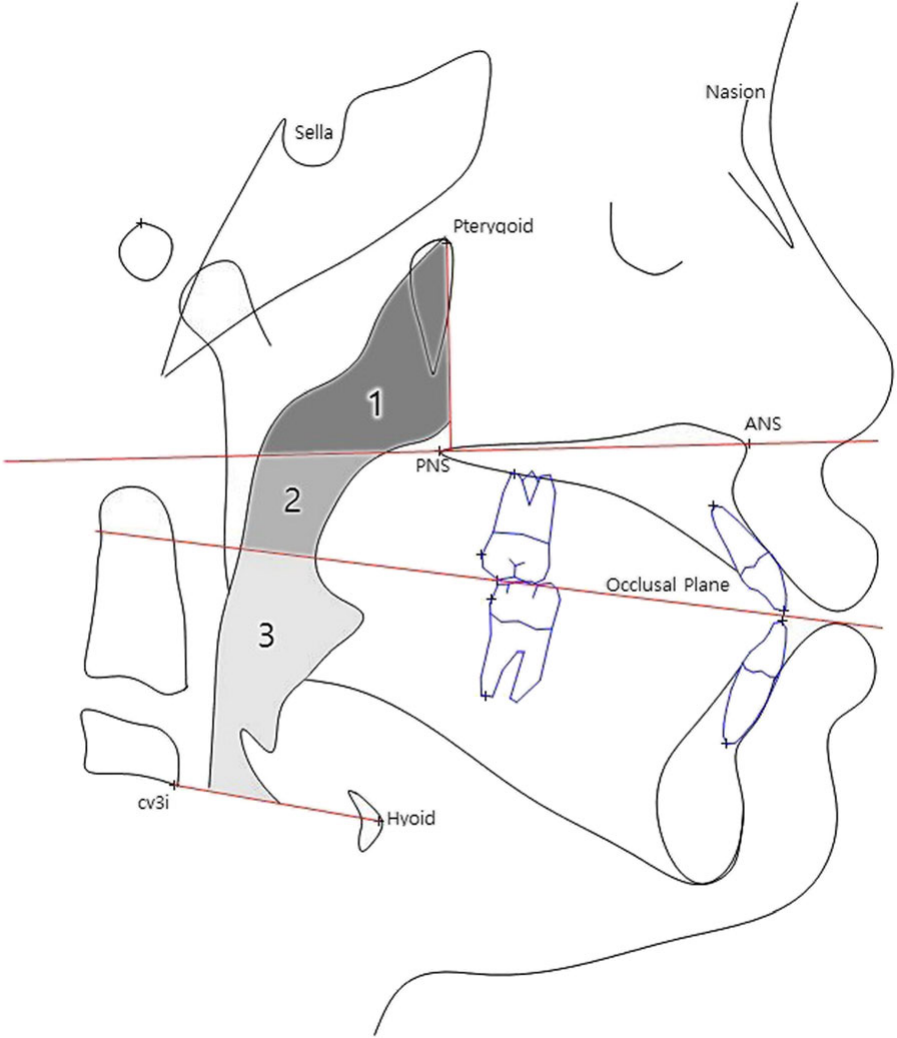
Anatomic points and areal measurements used in this study. *Cv3i* indicates the most inferior point of the third cervical vertebra, *Hyoid* the most superior and anterior point on body of the hyoid bone. *1* SPPA, *2* MPA, *3* IPA

Lateral cephalograms were taken before treatment (T1) and after treatment (T2) and traced by a single investigator. Figure 3 shows cephalometric measurements and maxillary advancement measurement (Co-A) is represented as a dotted line. Figures 4 and 5 describe reference points and cephalometric measurements of the pharyngeal airway. Specific measurements used in this study are categorized into linear measurement and areal measurement as follows:

1. Linear measurements (Fig. 4)
  1. Superior pharyngeal space (SPPS): The width of the pharynx measured between the posterior pharyngeal wall and the dorsum of the soft palate on a line parallel to the FH plane (the line through Po and Or) that runs through the middle of the line from PNS to P
  2. Middle pharyngeal space (MPS): The width of the pharynx measured between the posterior pharyngeal wall and the dorsum of the tongue on a line parallel to the FH plane that runs through P
  3. Inferior pharyngeal space (IPS): The width of the pharynx measured between the posterior pharyngeal wall and the dorsum of the tongue on a line parallel to the FH plane that runs through C2i
2. Areal measurements (Fig. 5)
  1. Superior pharyngeal area (SPPA): The area of the pharynx with an inferior border on the ANS-PNS extension line and an anterior border on the line perpendicular to the ANS-PNS line that runs through the pterygoid
  2. Middle pharyngeal area (MPA): The area of the pharynx with a superior border on the ANS-PNS extension line and an inferior border of the extended occlusal plane
  3. Inferior pharyngeal area (IPA): The area of the pharynx with a superior border on the extended occlusal plane and an inferior border of the most anteroinferior point on the body of the third cervical vertebra (cv3i)—the most anterior point of the hyoid bone (hy) line

The length of linear measurements and area of areal measurements were calculated with V-Ceph (version 7.0, Osstem, Seoul, Korea).

### Statistical analysis

Normality evaluation of the TBFM and SAFM groups between T1 and T2 was done by the Kolmogorov-Smirnov test. At the time of T1, the differences between the TBFM and SAFM groups were evaluated by an independent *t* test. Also, for each group, changes in measurements were evaluated between T1 and T2 using an independent *t* test. The independent *t* test and Mann-Whitney *U* test were performed to compare the changes in both groups after treatment (T2). The normality test showed that changes in MPS, IPS, and IPA were parametric, but changes in SPPS, SPPA, and MPA were non-parametric. So, independent *t* tests were performed to compare changes in MPS, IPS, and IPA between the TBFM group and SAFM group and Mann-Whitney *U* tests were performed to compare changes in SPPS, SPPA, and MPA between the TBFM group and the SAFM group. Data from the four subgroups was not normally distributed because of the small sample size. So, the Kruskal-Wallis test was employed to assess differences among subgroups in pharyngeal airway measurements. If any differences between the subgroups were found in linear or areal measurements, the Mann Whitney *U* test was used to evaluate those differences. Statistical analysis was done using IBM SPSS Statistics (version 21.0, IBM, New York) at a 0.05 significance level.

Five patients from each group were chosen randomly for a re-evaluation after 4 weeks. A total of ten cephalograms were traced again and areal measurements and linear measurements were re-calculated by the same researcher. The original measurements and measurements after 4 weeks were analyzed by Cohen’s kappa. The coefficient of Cohen’s kappa for the areal measurements was 0.92, and the coefficient for linear measurements was 0.95.

## Results

At time T1, both the TBFM and SAFM groups were normally distributed, so an independent *t* test was performed to evaluate the difference between the groups.

Table 1 shows the comparison between the TBFM and SAFM groups at time T1 (Table 1). Both groups showed characteristics of class III malocclusion, retrusion of maxilla, and relatively protrusion of mandible. There were no statistically significant differences in cephalometric measurements and pharyngeal airway measurements between the groups before treatment. Since the purpose of this study was analysis of pharyngeal airway space, the following context focused on the change of pharyngeal airway measurements.

**Table 1:** Comparison of cephalometric measurements and pharyngeal airway measurements between tooth-borne face mask (TBFM) and skeletal anchored face mask (SAFM) patients before treatment. Normal range of pharyngeal airway measurement were not included

	Normal data		TBFM		SAFM		*P*
	Mean	SD	Mean	SD	Mean	SD	
SNA	81.80	2.82	79.33	2.94	79.53	3.23	0.156
SNB	77.00	2.82	81.02	3.22	80.78	2.96	0.630
N. per. to A	0.40	2.30	−3.02	2.14	−4.37	2.54	0.190
Palatal P.	1.20	4.72	0.74	2.32	0.45	2.42	0.561
Co-A	91.00	4.30	82.61	3.96	81.95	4.32	0.854
Mn. length	113.12	3.62	112.21	4.51	114.19	4.83	0.259
ANB	3.00	1.82	−1.22	1.89	−2.22	2.15	0.094
U1 to SN	105.82	5.82	108.18	8.49	109.09	7.41	0.837
FMA	25.00	2.00	27.91	2.81	27.82	5.24	0.912
IMPA	88.00	2.00	87.72	6.73	85.32	5.51	0.176
SPPS			11.46	2.20	11.72	2.26	0.680
MPS			12.26	3.35	13.12	2.68	0.323
IPS			12.56	2.17	11.74	3.26	0.232
SPPA			248.32	52.59	265.14	58.84	0.287
MPA			190.70	23.61	198.70	42.82	0.406
IPA			298.22	60.32	371.97	74.43	0.445

Independent *t* test, *P* < 0.05

*SD* standard deviation

At time T2, both groups had increased pharyngeal airway measurements in comparison with time T1. The changes in airway measurements are shown in Tables 2 and 3. In the TBFM group, SPPS, SPPA, and MPA at T2 were significantly greater than those at T1, whereas MPS, IPS, and IPA at time T2 were not significantly different (Table 2). However, the SPPS, MPS, IPS, SPPA, and MPA of the SAFM group were significantly increased at time T2. Only the IPA of the SAFM group showed no significant increase after treatment.

**Table 2:** Airway measurements before before (T1) and after treatment (T2) treatment with tooth-borne facemask (TBFM)

			SPPS	MPS	IPS	SPPA	MPA	IPA
TBFM	T1	Mean	11.46	12.26	12.56	248.32	190.70	298.22
		SD	2.19	3.35	2.17	52.59	23.61	56.76
	T2	Mean	12.78	13.68	13.67	285.29	218.99	324.86
		SD	2.01	2.97	2.07	64.91	33.60	65.19
*P*			0.022	0.099	0.056	0.023	0.001	0.109

Independent *t* test, *P* < 0.05; measurements are in millimeters

*SD* standard deviation

**Table 3:** Airway measurements before (T1) and after (T2) treatment with skeletal anchored face mask (SAFM)

			SPPS	MPS	IPS	SPPA	MPA	IPA
SAFM	T1	Mean	11.72	13.12	11.74	265.14	198.70	310.08
		SD	2.31	2.73	2.70	60.11	43.74	53.68
	T2	Mean	14.24	15.18	14.28	341.24	229.15	382.14
		SD	3.59	3.35	3.81	70.94	68.68	75.34
*P*			0.006	0.006	0.024	0.011	0.000	0.075

Independent *t* test, *P* < 0.05; measurements are in millimeters

*SD* standard deviation

Table 4 summarizes changes in pharyngeal airway measurements of the TBFM group and the SAFM group throughout facemask treatment. All linear and areal measurements of both groups increased at time T2 compared to T1.

**Table 4:** Changes in airway measurements after tooth-borne face mask (TBFM) and skeletal anchored face mask (SAFM) therapy

		∆SPPS	∆MPS	∆IPS	∆SPPA	∆MPA	∆IPA
TBFM	Mean	1.32	1.81	1.11	36.97	28.30	26.64
	SD	1.13	1.05	1.55	38.34	30.53	42.21
SAFM	Mean	2.53	2.81	2.54	76.10	30.44	72.06
	SD	3.13	1.63	2.11	59.32	46.80	54.76
*P*		0.173	0.014	0.007	0.002	0.935	0.001
			*	**	**		**

Measurements are in millimeters

*SD* standard deviation

*P < 0.05; **P < 0.01

### Linear measurements

Among the linear measurements, SPPS was not normally distributed. SPPS of both groups increased after facemask treatment, but not in a statistically significant manner (*P* > 0.05). The MPS and IPS of the TBFM and SAFM groups were normally distributed, and both measurements in the SAFM group were significantly greater than those in the TBFM group (MPS *P* < 0.05; IPS *P* < 0.01).

### Areal measurements

Among the areal measurements, SPPA and MPA were not normally distributed, whereas IPA was normally distributed. The SPPA of the SAFM group was increased significantly more than the SPPA of the TBFM group (*P* < 0.01). The MPA after face mask treatment was increased in both groups; however, there were no significant differences between the two groups (*P* > 0.05). The IPA of the SAFM group was significantly greater than that of the TBFM group after treatment (*P* < 0.01).

Table 5 shows the mean values of maxillary advancement after face mask therapy in both groups. In both Co-A and SNA, the mean values of the SAFM group were greater than those of the TBFM group, but the differences were not statistically significant (*P* > 0.05).

**Table 5:** Mean values of maxillary advancement after face mask therapy

Maxillary advancement		∆ Co-A (mm)	∆ SNA (°)
TBFM	Mean	2.74	1.76
	SD	1.73	1.03
SAFM	Mean	3.63	2.39
	SD	1.78	1.15
*P*		0.081	0.165

Independent *t* test, *P* < 0.05

*SD* standard deviation

In the TBFM group, the mean value of maxillary advancement was 2.74 mm. The subgroups were divided on the basis of this value. Thirteen subjects (5 boys and 8 girls) who showed maxillary protraction more than 2.74 mm were included in TBFM subgroup A and 15 subjects (3 boys and 12 girls) whose maxillary protraction was less than 2.74 mm were included in TBFM subgroup B. The mean value of maxillary protraction in the SAFM group was 3.63 mm, and subgrouping was done in the same way. Thirteen subjects (3 boys and 10 girls) were included in SAFM subgroup A and 11 subjects (9 boys and 2 girls) were included in SAFM subgroup B.

### Analysis of subgroups

Subgroup analysis of each airway measurement is shown in Tables 6 and 7. The four subgroups showed statistical differences in SPSS on the Kruskal-Wallis test (*P* < 0.05). A Mann-Whitney *U* test then showed that the increase in SPPS was statistically greater in SAFM subgroup A than in TBFM subgroup B. There were no significant differences between other subgroups. In terms of MPS, IPS, and MPA, no significant differences were found between the subgroups. There were significant differences in SPPA and IPA among the subgroups (*P* < 0.05). A Mann-Whitney *U* test revealed that both SAFM subgroups showed significantly greater increases in SPPA and IPA than either TBFM subgroup. There were no significant differences between the two TBFM subgroups or between the two SAFM subgroups.

**Table 6:** Mean values of each subgroup and Kruskal-Wallis test results of airway measurements

Kruskal-Wallis test		∆ SPPS	∆ MPS	∆ IPS	∆ SPPA	∆ MPA	∆ IPA
TBFM	Subgroup A	1.54	1.88	1.02	35.6	30.8	24.13
	Subgroup B	1.12	1.78	1.26	36.99	26.37	27.88
SAFM	Subgroup A	3.74	2.91	2.50	64.65	34.34	72.37
	Subgroup B	1.09	2.68	2.58	89.61	25.82	71.68
*P*		0.035*	0.092	0.078	0.014*	0.982	0.006*

Measurements are in millimeters

*P < 0.05

**Table 7:** Mann-Whitney *U* test results following Kruskal-Wallis test

Mann-Whitney *U* test		SAFM subgroup A − SAFM subgroup B	SAFM subgroup A − TBFM subgroup A	SAFM subgroup A − TBFM subgroup B	SAFM subgroup B − TBFM subgroup A	SAFM subgroup B − TBFM subgroup B	TBFM subgroup A − TBFM subgroup B
*P*	SPPS	0.223	0.112	0.019*	0.601	0.058	0.053
	SPPA	0.459	0.043*	0.029*	0.013*	0.021*	0.618
	IPA	0.955	0.007*	0.010*	0.015*	0.020*	0.555

*P < 0.05

## Discussion

Facemask therapy induces forward displacement of the maxilla and decreases forward displacement of the mandible. This is accomplished by stimulating cellular activity in circummaxillary sutures and the maxillary tubercle [19, 20]. Although conventional facemask therapy has proven to be an efficient method for treating skeletal class III malocclusion, because of unwanted dentoalveolar effects of conventional tooth-borne face masks, there have been numerous attempts to develop a method of skeletal face mask anchorage. Kokich et al. used an intentionally ankylosed deciduous tooth as an anchor point, Singer et al. and Enacar et al. introduced osseointegrated implants as anchors, Hong et al. placed a hexagonal implant in the palatal bone and used this as an anchor point, Kircelli and Pektas inserted two miniplates in the lateral nasal wall of the maxilla and placed elastics in the hooks of the miniplates, and De Clerck et al. used two miniplates inserted in the infrazygomatic crest and two miniplates inserted between the canine and the first premolar in the mandible as anchors [12, 21–25].

In SAFM protocol, there are some controversies about proper magnitude of maxillary protraction force. Nguyen et al. [18]. applied initial force of 100 g on each side and increased force up to 200 g after 1 month. But Sar et al. [13] delivered protraction force of 400 g on each side and acquired proper maxillary advancement. Some clinicians think that elastic force of 400 g on each side is insufficient and need even more force for adequate maxillary protraction, but there is lack of substantial studies about proper protraction force.

The relationship between facemask therapy and airway volume increase has been widely investigated [16, 17, 26, 27]. However, controversy remains concerning the effectiveness of facemask therapy on airway dimension increases. Hiyama et al. and Kaygisiz et al. reported that pharyngeal airways improved after correcting maxillary retrusion by facemask therapy [16, 17]. On the other hand, Baccetti et al. and Mucedero et al. found that there were no significant increases in pharyngeal airway volume after face mask therapy [26, 27]. In the present study, we observed increases in pharyngeal airway volume after treatment in both groups of patients.

This study used a modification of Kircelli’s miniplate anchorage system because of its relative simplicity and effectiveness. Conventional miniplates used to treat trauma patients were initially employed, but screw loosening and plate detachment from the maxilla happened frequently because of the bone flexibility in young patients. So, the fixation system was changed to a locking system in order to increase stability and make screw loosening less likely. However, our plate and screw fixation method still has some disadvantages. Although we used locking fixation system, there was one patient who showed loosening of miniplate and consequently needed removal of miniplate and re-operation was performed just lateral to former position. According to Sar et al. [13], loosening of miniplate were up to 7% and in that case, the miniplate should be replaced. In addition to screw loosening, these miniplates were not designed for skeletal anchorage, they need to be modified preoperatively. Another disadvantage involves the hook at the last hole. This hook has internal threads for the locking system. Although surgeons smooth the threads before placement, some sharp edges remain, which can cause the elastics to break. These problems originate from the fact that we used miniplates designed for trauma or orthognathic surgery. If this skeletal anchorage method gains popularity, a plate and screw system optimized for this purpose could be created.

Changes in airway measurements were assessed using lateral cephalometric radiographs. Although lateral cephalograms can only provide two-dimensional information, they have been used to efficiently analyze pharyngeal airway spaces. Not only are they simple and easy to use but there is also a significant correlation between airway space measured with a lateral cephalogram and measured with computed tomography according to Riley and Powell [28]. Various studies have used lateral cephalometric films to investigate pharyngeal airway changes. It is widely accepted that there is a correlation between head posture and pharyngeal airway volume [16, 29]. Therefore, all lateral cephalometric radiographs should be taken with the head in a natural position, and the cephalometric films used in this study were taken following standard rules.

One limitation of this study is that it lacks a control (untreated) group. Comparison of linear and areal measurements between a control group and TBFM and SAFM groups would be ideal. However, in order to acquire cephalometric radiographs of control subjects, patients with skeletal class III malocclusion would have to be left untreated, which causes an ethical dilemma. Also, the study involves exposure to radiation, which is another ethical issue. Fortunately, there is precedence for carrying out studies of this nature without an untreated control group.

Airway passages usually expand as individuals grow up. Some authors have conducted research on the correlation between physical growth and pharyngeal airway volume [30–34]. According to Taylor et al., most posterior pharyngeal wall growth occurred in two spurts, from 6 to 9 years of age and from 12 to 15 years of age; growth of airways in individuals 9 to 12 years old is very limited [32]. The subjects in the current study consisted of 20 boys and 32 girls, with a mean age of 10.7 years and a mean treatment time of 15.5 months. Therefore, we can assume that the extent of natural pharyngeal airway growth in these subjects would be insignificant and the airway increases we observed was induced by face mask treatment.

Both the TBFM and SAFM groups showed increased pharyngeal airway measurements after treatment, but the SAFM group showed statistically significantly greater increases than did the TBFM group in four measurements. The SAFM subgroups had greater SPPA and IPA increases than did the TBFM subgroups. These findings suggest that SAFM therapy is more effective than TBFM therapy for increasing airway volume.

It is uncertain why the upper airway space increased by maxillary protraction. Some possible explanations are as follows. First, the protraction force of the face mask may induce forward movement of the maxilla, especially PNS; this could cause anterior displacement of the soft palate and consequently increase upper airway space [16]. Second, the anterior position of the tongue is altered by facemask treatment. This may be induced by increased volume of the oral cavity or by clockwise rotation of the mandible. The altered tongue posture could lead to an anterior shift of the soft palate and increased upper airway space [35].

Recently, pediatric obstructive sleep apnea (OSA) has attracted a great deal of attention. Many treatment modalities are available for pediatric OSA. Some authors proposed that maxillary protraction can be a solution for OSA [36]. Hiyama et al. [16] suggested that maxillary protraction appliances could contribute to improvements in respiratory function in patients with maxillary hypoplasia. Similarly, Hüsamettin et al. [37] implied that maxillary protraction could alleviate respiratory discomfort in patients with maxillary retrusion, and Verse et al. [38] found that intraoral devices were effective in approximately 50–70 percent of patients with obstructive sleep apnea. Conley [39] evaluated the effects of dental treatment in pediatric patients with obstructive sleep apnea. According to that study, maxillary protraction with palatal expansion has the potential to treat pediatric OSA patients. However, there are still an insufficient number of studies on the effects of maxillary protraction on treatment outcomes of OSA, such as measurements of polysomnography. Given the results of the current study, increased upper airway dimensions resulting from use of SAFM could contribute to the treatment of pediatric OSA. Unfortunately, none of our subjects reported a history of OSA, so further studies using specific measurements of OSA are needed in order to evaluate SAFM as a treatment for OSA.

## Conclusions

There were significant increases in upper airway space in the TBFM and SAFM groups after maxillary protraction. The SAFM group exhibited greater overall pharyngeal airway increases than did the TBFM group, and SAFM subgroups showed greater increases in airway measurement values than did the TBFM subgroups, especially measurements of SPPA and IPA. Thus, SAFM was more effective than TBFM in producing airway increases. SAFM treatment may improve respiratory function in patients with maxillary hypoplasia or OSA, but this hypothesis requires further investigations.

To our knowledge, there has been no previous report comparing pharyngeal airway changes between TBFM and SAFM treatment. Therefore, we can say this study has produced meaningful results, even though it lacks data on untreated subjects.

## References

[1] Nanda R (1980). Biomechanical and clinical considerations of a modified protraction headgear. Am J Orthod.

[2] Ellis E, McNamara JA (1984). Components of adult class III malocclusion. J Oral Maxillofac Surg.

[3] Guyer EC, Ellis EE, McNamara JA, Behrents RG (1986). Components of class III malocclusion in juveniles and adolescents. Angle Orthod.

[4] Haynes S. (1970) The prevalence of malocclusion in English children aged 11-12 years. Rep Congr Eur Orthod Soc 89–98 5287526

[5] Thilander B, Myrberg N (1973). The prevalence of malocclusion in Swedish schoolchildren. Scand J Dent Res.

[6] Ishii H, Morita S, Takeuchi Y, Nakamura S (1987). Treatment effect of combined maxillary protraction and chincap appliance in severe skeletal class III cases. Am J Orthod Dentofacial Orthop.

[7] McNamara JA (1987). An orthopedic approach to the treatment of class III malocclusion in young patients. J Clin Orthod.

[8] Proffit WR (1993). Contemporary orthodontics.

[9] Nanda R (1978). Protraction of maxilla in rhesus monkeys by controlled extraoral forces. Am J Orthod.

[10] Kircelli BH, Pektas ZO, Uckan S (2006). Orthopedic protraction with skeletal anchorage in a patient with maxillary hypoplasia and hypodontia. Angle Orthod.

[11] Zhou YH, Ding P, Lin Y, Qiu LX (2007). Facemask therapy with miniplate implant anchorage in a patient with maxillary hypoplasia. Chin Med J (Engl).

[12] Kircelli BH, Pektas ZO (2008). Midfacial protraction with skeletally anchored face mask therapy: a novel approach and preliminary results. Am J Orthod Dentofacial Orthop.

[13] Sar C, Arman-Ozcirpici A, Uckan S, Yazici AC (2011). Comparative evaluation of maxillary protraction with or without skeletal anchorage. Am J Orthod Dentofacial Orthop.

[14] Koh SD, Chung DH (2014). Comparison of skeletal anchored facemask and tooth-borne facemask according to vertical skeletal pattern and growth stage. Angle Orthod.

[15] Sar C, Sahinoglu Z, Ozcirpici AA, Uckan S (2014). Dentofacial effects of skeletal anchored treatment modalities for the correction of maxillary retrognathia. Am J Orthod Dentofacial Orthop.

[16] Hiyama S, Suda N, Ishii-Suzuki M, Tsuiki S, Ogawa M, Suzuki S (2002). Effects of maxillary protraction on craniofacial structures and upper-airway dimension. Angle Orthod.

[17] Kaygisiz E, Tuncer BB, Yuksel S, Tuncer C, Yildiz C (2009). Effects of maxillary protraction and fixed appliance therapy on the pharyngeal airway. Angle Orthod.

[18] Nguyen T, De Clerck H, Wilson M, Golden B (2015). Effect of class III bone anchor treatment on airway. Angle Orthod.

[19] Kambara T (1977). Dentofacial changes produced by extraoral forward force in the Macaca irus. Am J Orthod.

[20] Jackson GW, Kokich VG, Shapiro PA (1979). Experimental and postexperimental response to anteriorly directed extraoral force in young Macaca nemestrina. Am J Orthod.

[21] Kokich VG, Shapiro PA, Oswald R, Koskinen-Moffett L, Clarren SK (1985). Ankylosed teeth as abutments for maxillary protraction: a case report. Am J Orthod.

[22] Singer SL, Henry PJ, Rosenberg I (2000). Osseointegrated implants as an adjunct to facemask therapy: a case report. Angle Orthod.

[23] Enacar A, Giray B, Pehlivanoglu M, Iplikcioglu H (2003). Facemask therapy with rigid anchorage in a patient with maxillary hypoplasia and severe oligodontia. Am J Orthod Dentofacial Orthop.

[24] Hong H, Ngan P, Han G, Qi LG, Wei SH (2005). Use of onplants as stable anchorage for facemask treatment: a case report. Angle Orthod.

[25] De Clerck HJ, Cornelis MA, Cevidanes LH, Heymann GC, Tulloch CJ (2009). Orthopedic traction of the maxilla with miniplates: a new perspective for treatment of midface deficiency. J Oral Maxillofac Surg.

[26] Mucedero M, Baccetti T, Franchi L, Cozza P (2009). Effects of maxillary protraction with or without expansion on the sagittal pharyngeal dimensions in class III subjects. Am J Orthod Dentofacial Orthop.

[27] Baccetti T, Franchi L, Mucedero M, Cozza P (2010). Treatment and post-treatment effects of facemask therapy on the sagittal pharyngeal dimensions in class III subjects. Eur J Orthod.

[28] Riley RW, Powell NB (1990). Maxillofacial surgery and obstructive sleep apnea syndrome. Otolaryngol Clin North Am.

[29] Pracharktam N, Hans MG, Strohl KP, Redline S (1994). Upright and supine cephalometric evaluation of obstructive sleep apnea syndrome and snoring subjects. Angle Orthod.

[30] Handelman CS, Osborne G (1976). Growth of the nasopharynx and adenoid development from one to eighteeen years. Angle Orthod.

[31] Linder-Aronson S, Leighton BC (1983). A longitudinal study of the development of the posterior nasopharyngeal wall between 3 and 16 years of age. Eur J Orthod.

[32] Taylor M, Hans MG, Strohl KP, Nelson S, Broadbent BH (1996). Soft tissue growth of the oropharynx. Angle Orthod.

[33] Preston CB, Tobias PV, Salem OH (2004). Skeletal age and growth of the nasopharynx in the sagittal plane: a cephalometric study. Semin Orthod.

[34] Jeans WD, Fernando DC, Maw AR, Leighton BC (1981). A longitudinal study of the growth of the nasopharynx and its contents in normal children. Br J Radiol.

[35] Ozbek MM, Memikoglu TU, Gogen H, Lowe AA, Baspinar E (1998). Oropharyngeal airway dimensions and functional-orthopedic treatment in skeletal class II cases. Angle Orthod.

[36] Gao X (2014). Pediatric sleep-disordered breathing and oral medicine. Hua xi kou qiang yi xue za zhi = Huaxi kouqiang yixue zazhi = West China journal of stomatology.

[37] Oktay H, Ulukaya E (2008). Maxillary protraction appliance effect on the size of the upper airway passage. Angle Orthod.

[38] Verse T, Pirsig W, Stuck BA, Hormann K, Maurer J (2003). Recent developments in the treatment of obstructive sleep apnea. Am J Respir Med.

[39] Conley RS (2011). Evidence for dental and dental specialty treatment of obstructive sleep apnoea. Part 1: the adult OSA patient and part 2: the paediatric and adolescent patient. J Oral Rehabil.

